# Death Not Always Painful

**Published:** 1854-12

**Authors:** Chas. Knowlton


### DEATH NOT ALWAYS PAINFUL.

WE think that most persons have been led to regard dying as a much more painful change than it generally is; first, because they have found by what they experienced in themselves and observed in others, that sentient beings often struggle when in distress; hence struggling to them is an invariable sign of distress. But we may remark, that struggles are very far from being an invariable sign of distress; muscular action and consciousness are two distinct things, often existing separately; and we have abundant reason to believe that in a great portion of cases, those struggles of dying man which are so distressing to behold, are as entirely independent of consciousness as the struggles of a recently decapitated fowl. A second reason why men are led to regard dying as a very painful change, is, because men often endure great pain without dying, and forgetting that like causes produce like effects only under similar circumstances, they infer that life cannot be destroyed without still greater pain. But the pains of death are much less than most persons have been led to believe, and we doubt not that many persons who live to the age of puberty, undergo tenfold more misery than they would, did they understand correct views concerning the change. In all cases of dying, the individual suffers no pain after the sensibility of his nervous system is destroyed, which is often without any previous pain.

Those who are struck dead by a stroke of lightning, those who are decapitated with one blow of the axe, and those who are instantly destroyed by a crush of the brain, experience no pain at all in passing from a state of life to a dead state. One moment's expectation of being thus destroyed far exceeds in misery the pain during the act. Those who faint in having a little blood taken from the arm, or on any other occasion, have already endured all the misery they ever would did they not again revive. Those who die of fevers and most other diseases, suffer their greatest pain, as a general thing, hours, or even days, before they expire. The sensibility of the nervous system becomes gradually diminished; their pain becomes less and less acute under the same existing cause; and at the moment when their friends think them in the greatest distress, they are more at ease than they have been for many days previous; their disease, as far as it respects their feelings, begins to act upon them like an opiate. Indeed, many are already dead as it respects themselves, when ignorant by-standers are much the most to be pitied, not for the loss of their friends, but their sympathizing anguish. Those diseases which destroy life without immediately affecting the nervous system, give rise to more pain than those that do affect the system so as to impair its sensibility. The most painful deaths which human beings inflict upon each other, are produced by rack and faggot. The halter is not so cruel as either of these, but more savage than the axe. Horror and pain considered, it seems to us that we should prefer a narcotic to either.—*Chas. Knowlton, M. D.*

